# Supplementary material for: Carborane-Containing Folic Acid bis-Amides: Synthesis and In Vitro Evaluation of Novel Promising Agents for Boron Delivery to Tumour Cells
Source: Int J Mol Sci. 2022 Nov 8;23(22):13726. doi: 10.3390/ijms232213726 (PMC9692863; doi:10.3390/ijms232213726)
Supplement: Supplementary file 1 [file ijms-23-13726-s001.zip › ijms-2014371-supplementary.pdf]

Supplementary Materials

Carborane-Containing Folic Acid *bis*-Amides: Synthesis and *In Vitro* Evaluation of Novel Promising Agents for Boron Delivery to Tumour Cells

Dmitry A. Gruzdev [1*], Angelina A. Telegina [1], Galina L. Levit [1], Olga I. Solovieva [2,3], Tatiana Ya. Gusel'nikova [3,4], Ivan A. Razumov [2,3], Victor P. Krasnov [1] and Valery N. Charushin [1,5]

[1] Postovsky Institute of Organic Synthesis, Russian Academy of Sciences (Ural Branch),Ekaterinburg 620108, Russia

[2] Institute of Cytology and Genetics, Russian Academy of Sciences (Siberian Branch),Novosibirsk 630090, Russia

[3] Novosibirsk State University, Novosibirsk 630090, Russia

[4] Nikolaev Institute of Inorganic Chemistry, Russian Academy of Sciences (Siberian Branch),Novosibirsk 630090, Russia

[5] Institute of Chemical Engineering, Ural Federal University, Ekaterinburg 620002, Russia

NMR Spectra

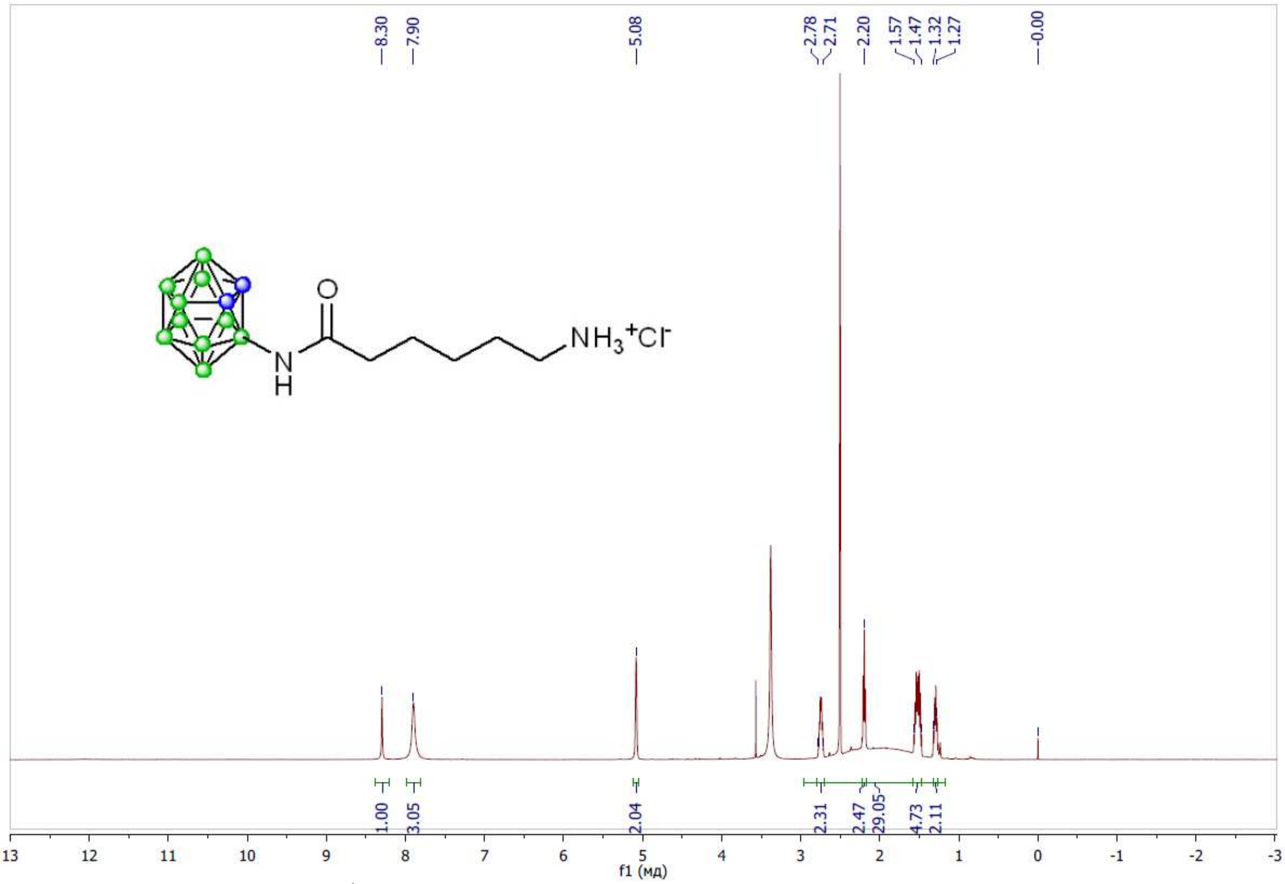

Figure S1. ^{1}H NMR spectrum of compound **1c** (DMSO-d_6, 500 MHz)

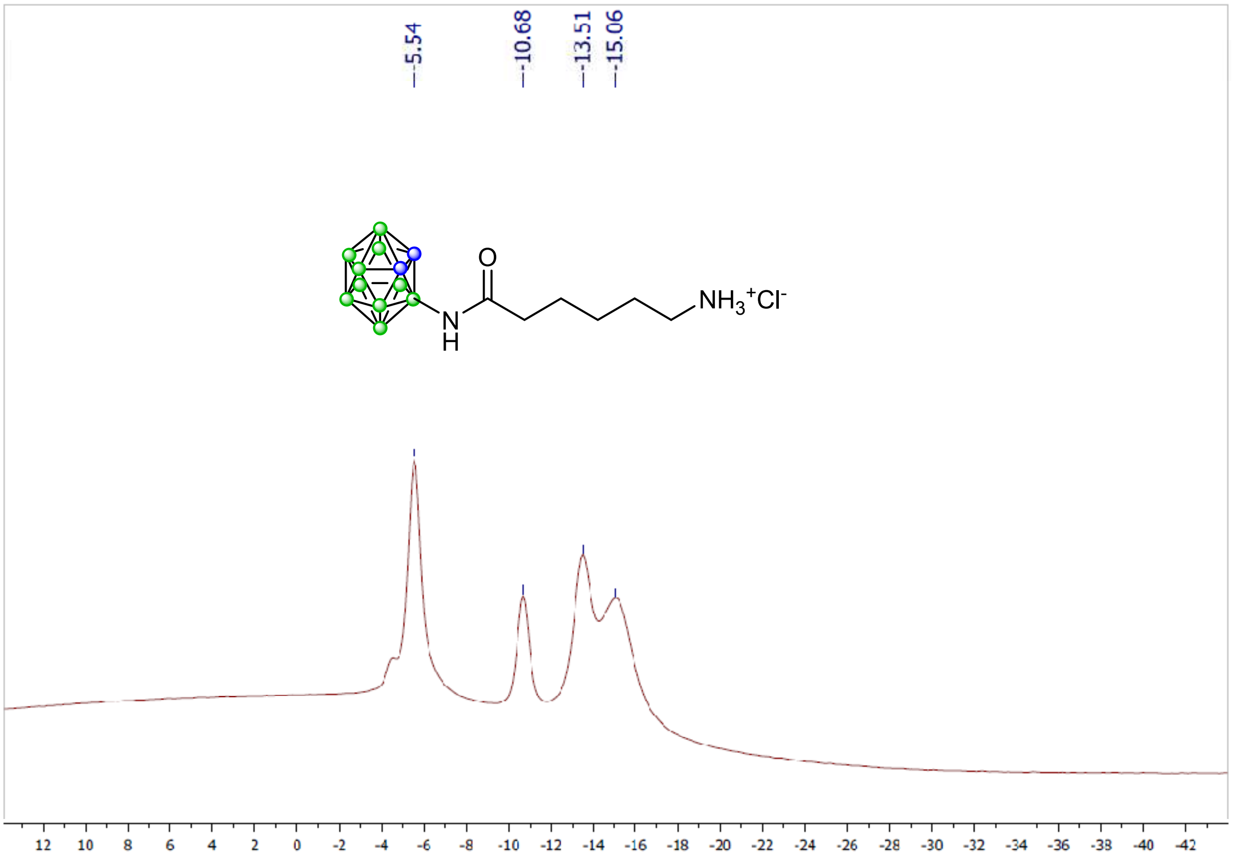

Figure S2. ^{11}B NMR spectrum of compound **1c** (DMSO-d_6, 160 MHz)

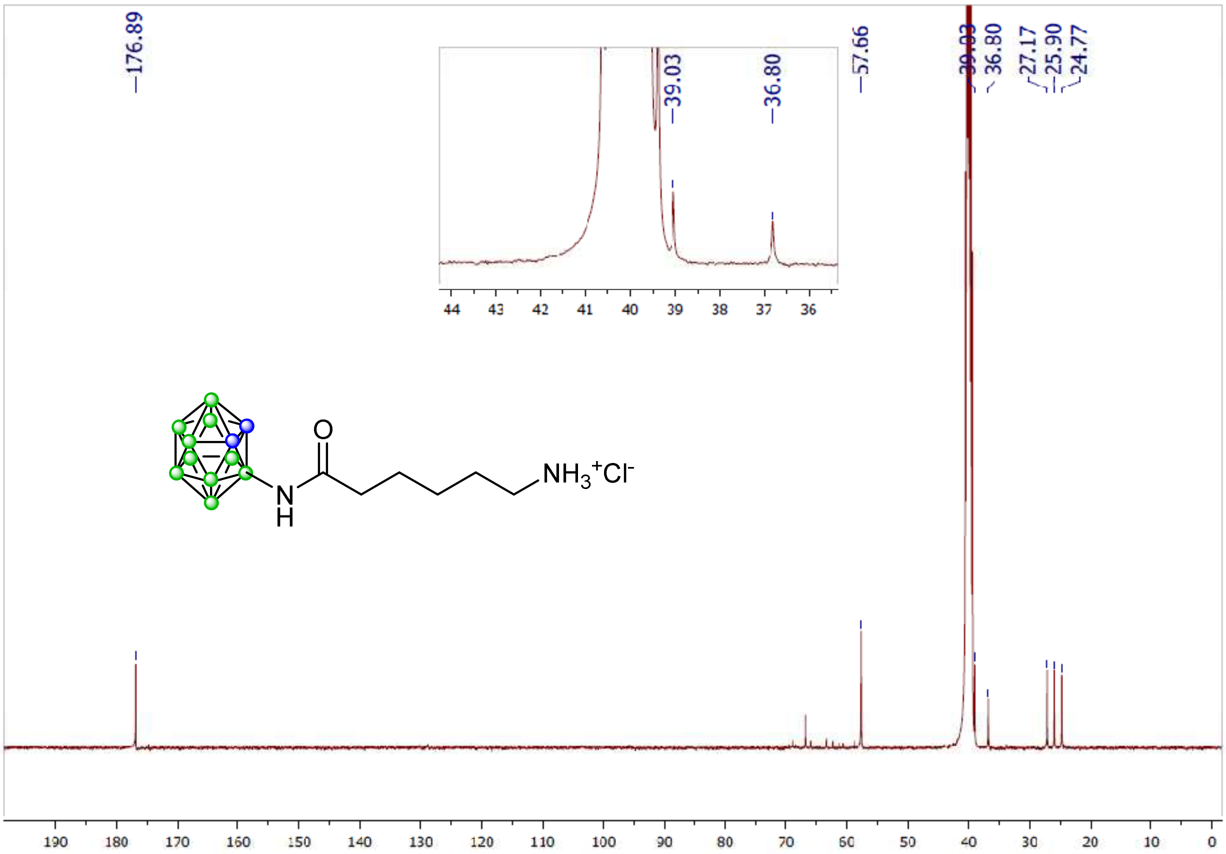

Figure S3. ^{13}C NMR spectrum of compound **1c** (DMSO-d_6, 100 MHz)

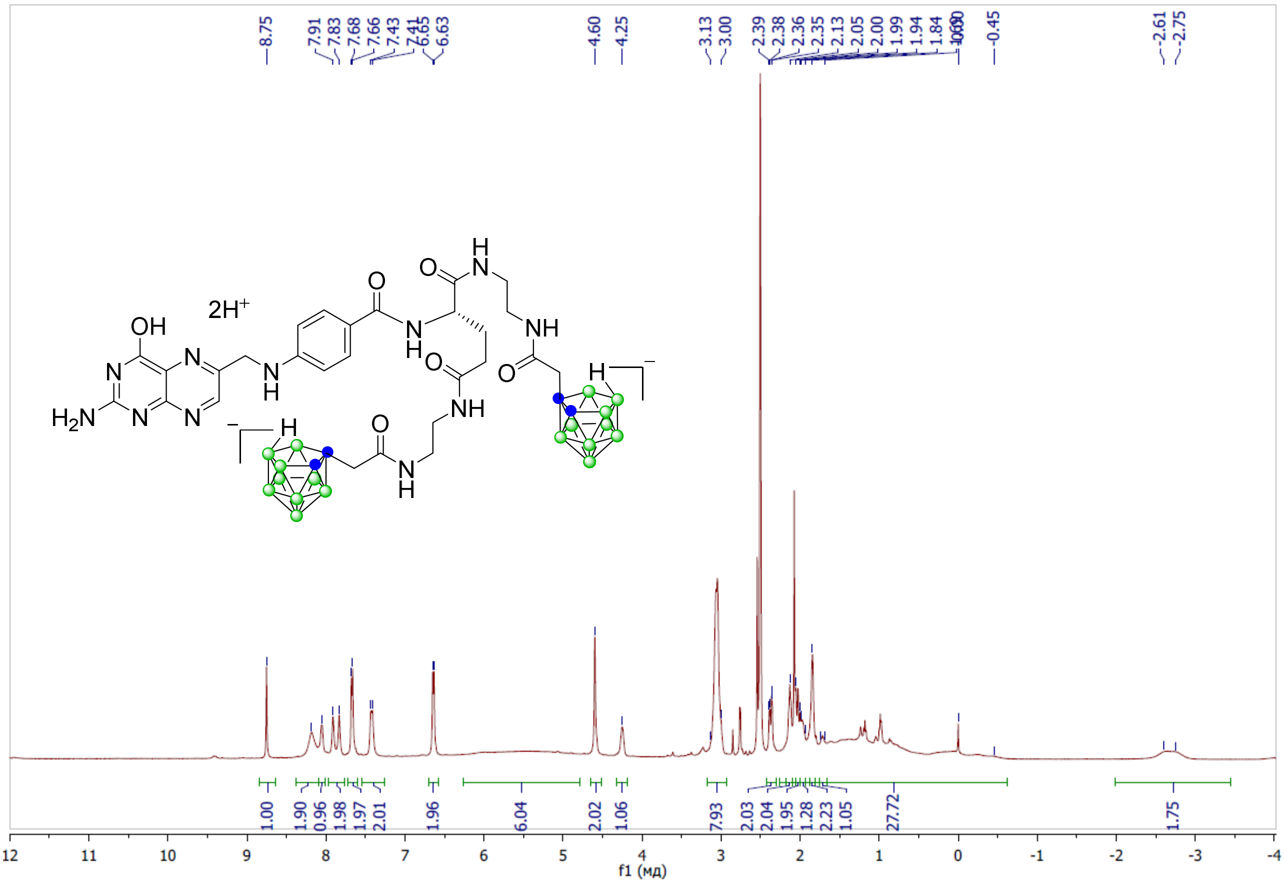

Figure S4. ^{1}H NMR spectrum of compound **3a** (DMSO-d_6, 500 MHz)

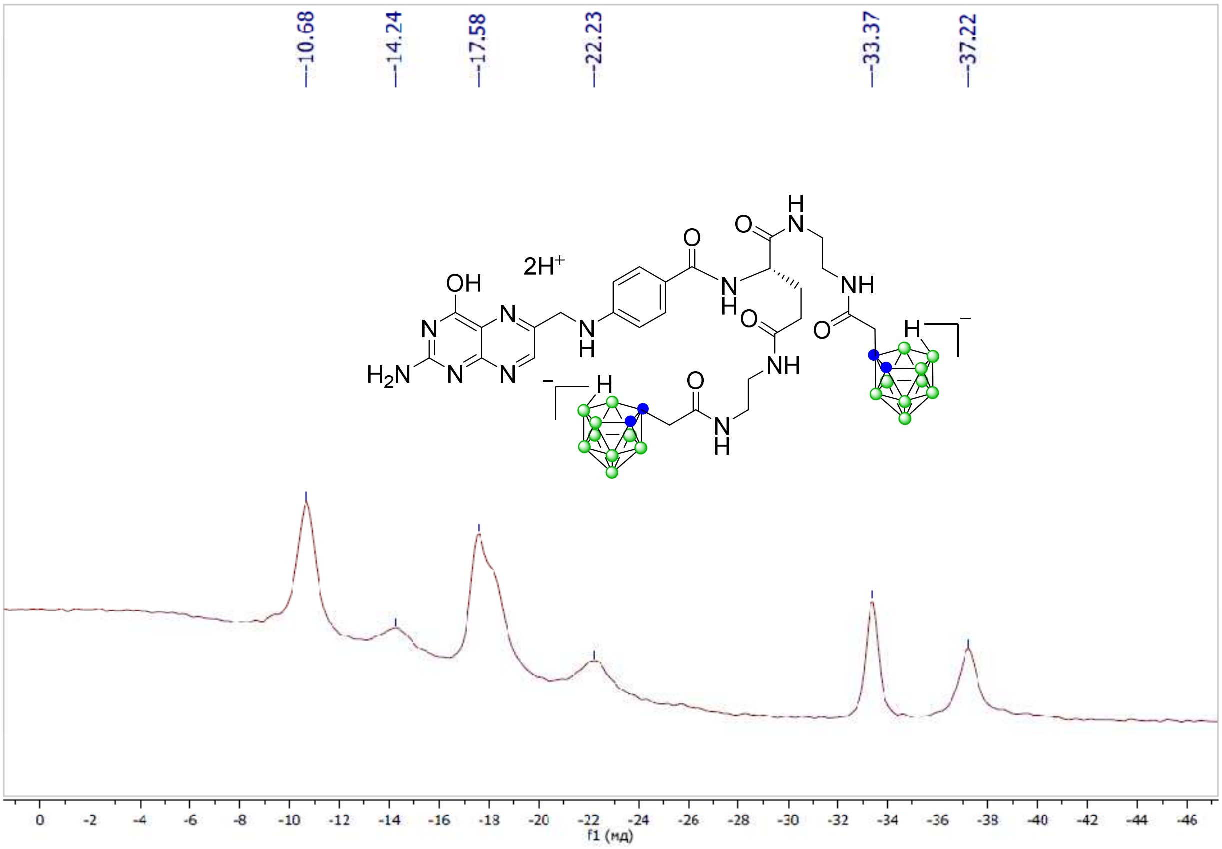

Figure S5. ^{11}B NMR spectrum of compound **3a** (DMSO-d_6, 160 MHz)

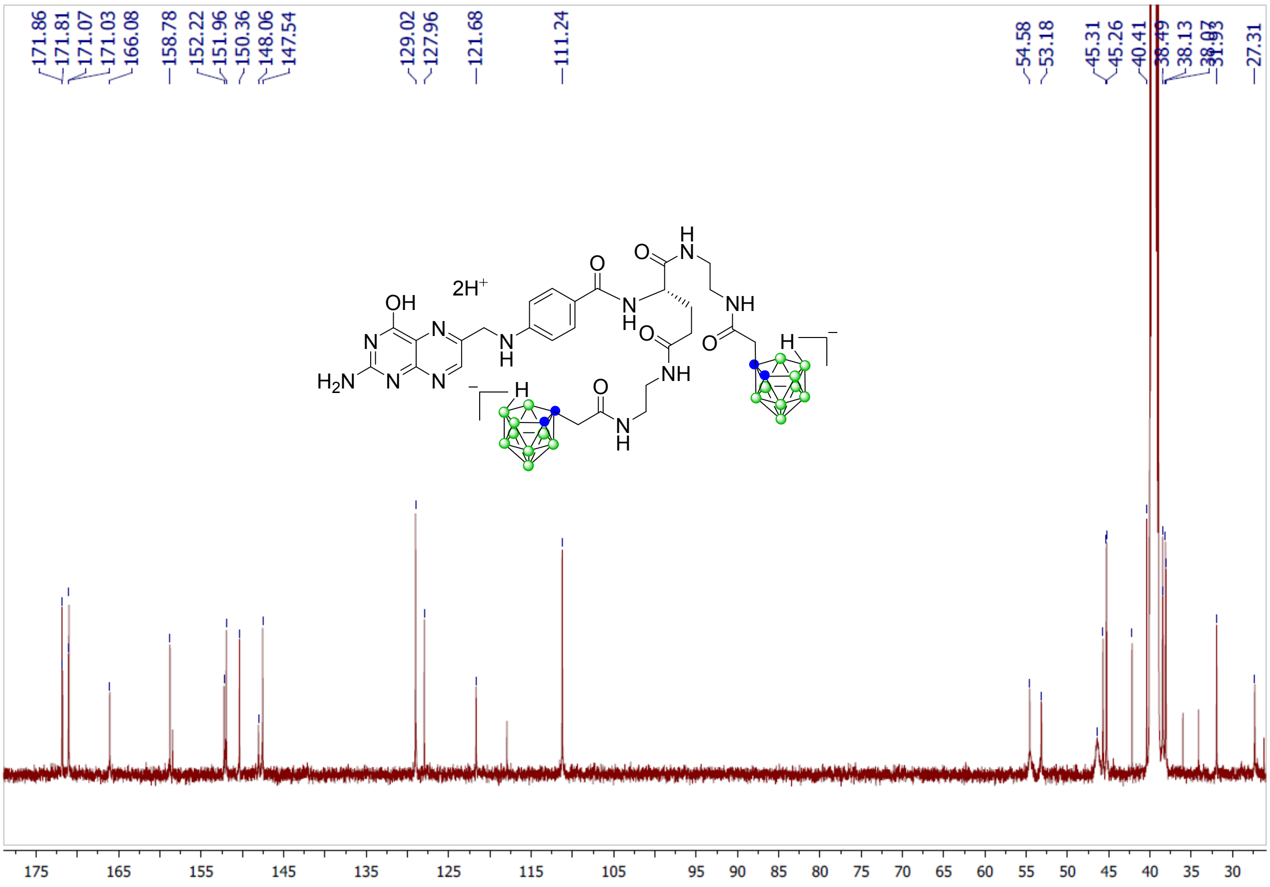

Figure S6. ^{13}C NMR spectrum of compound **3a** (DMSO-d_6, 126 MHz)

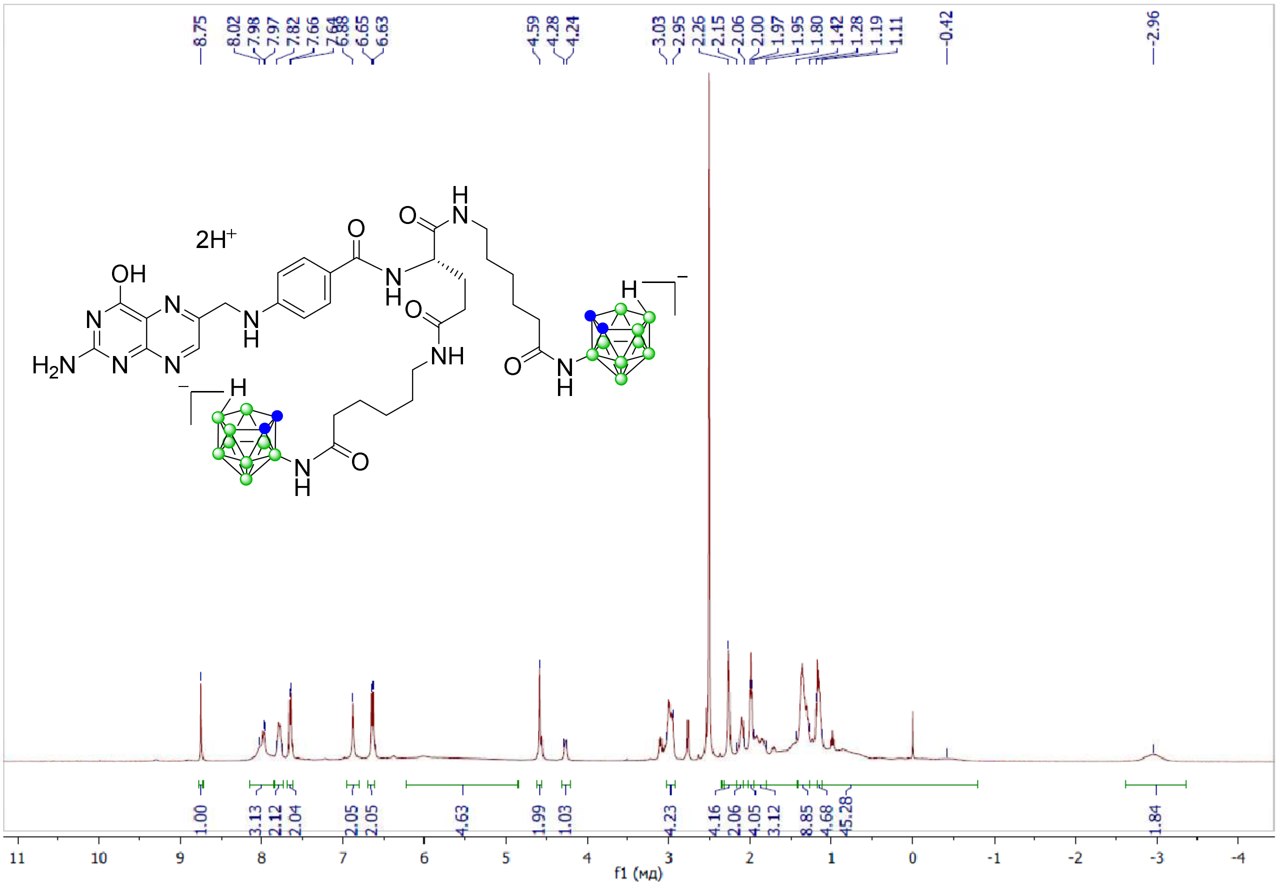

Figure S7. ^{1}H NMR spectrum of compound **3b** (DMSO-d_6, 500 MHz)

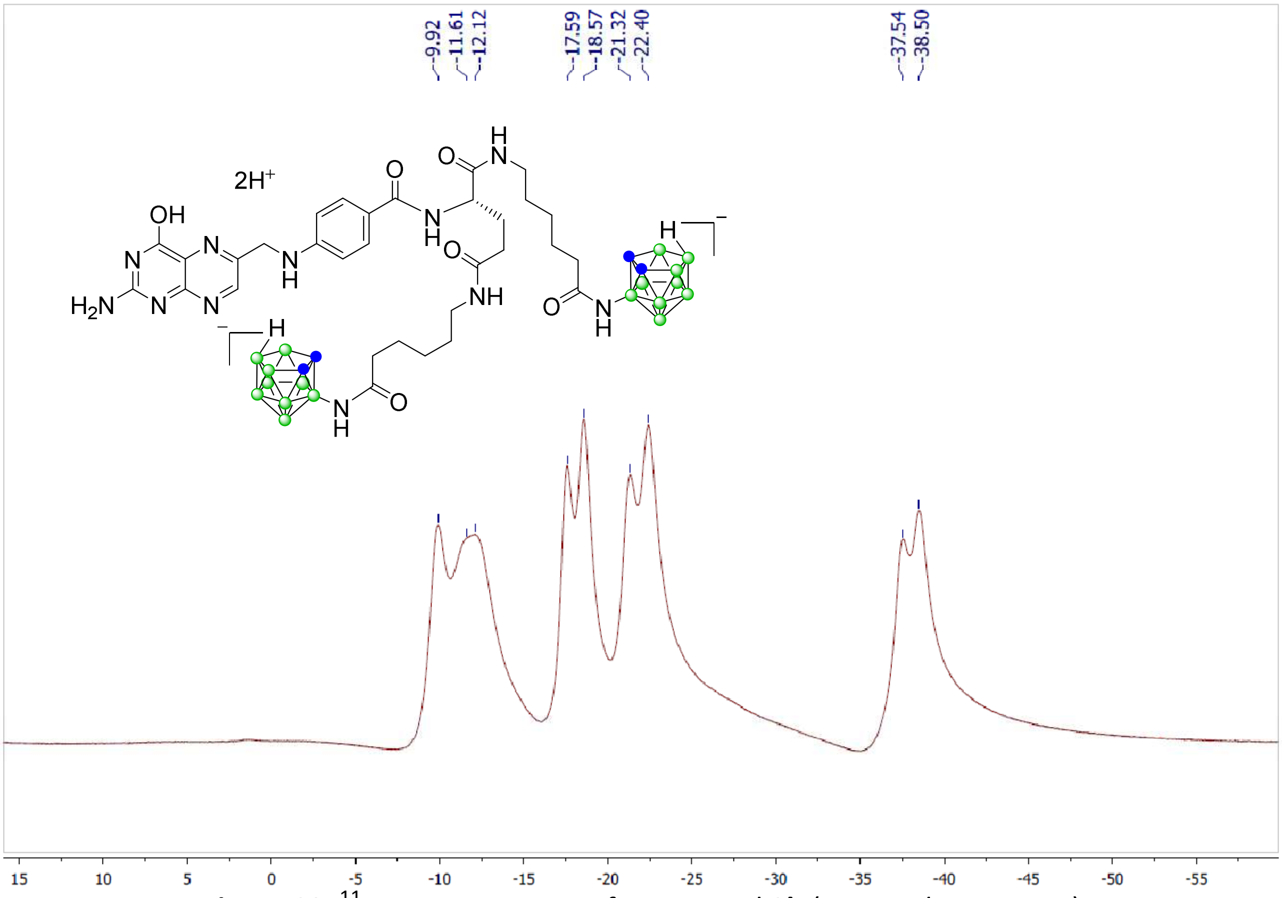

Figure S8. ^{11}B NMR spectrum of compound **3b** (DMSO-d_6, 128 MHz)

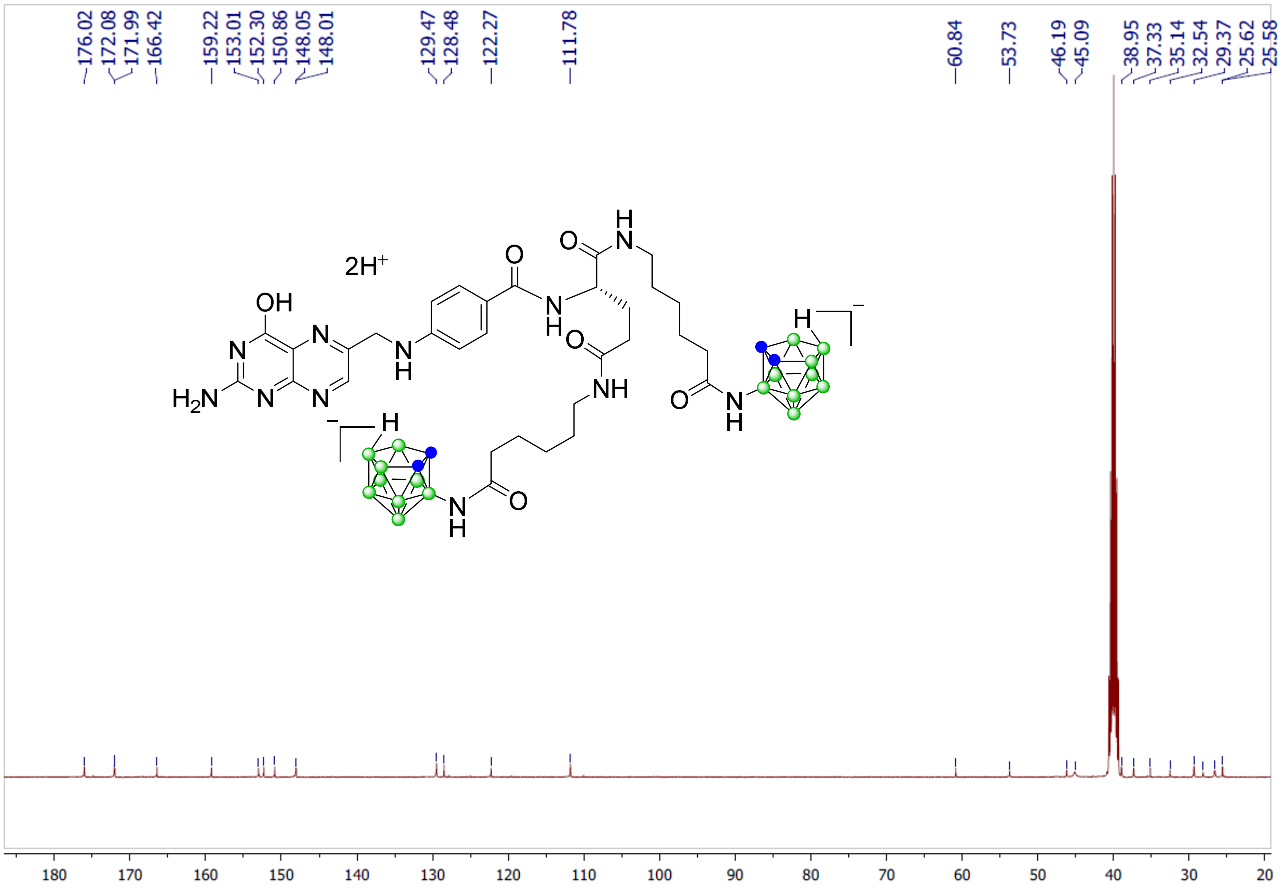

Figure S9. ^{13}C NMR spectrum of compound **3b** (DMSO-d_6, 100 MHz)

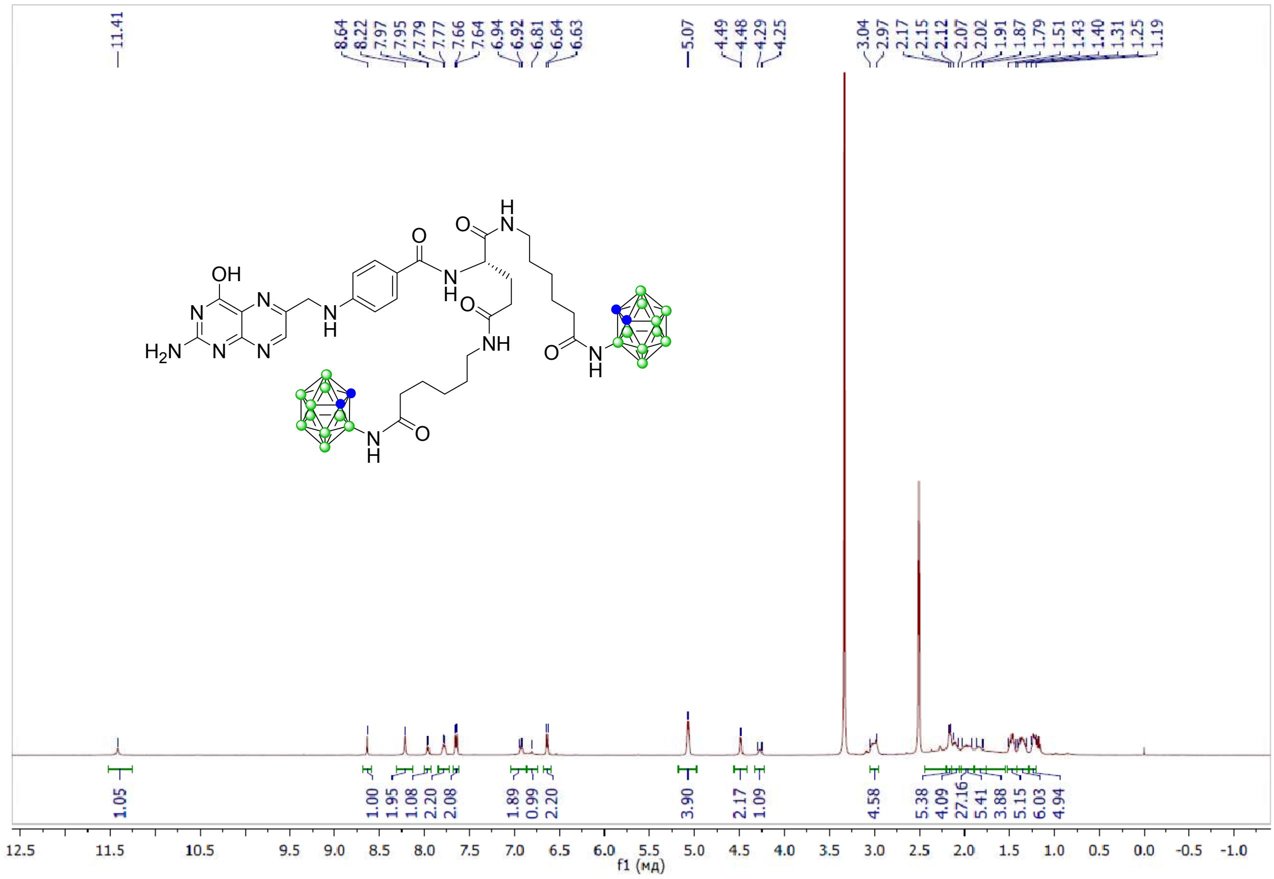

Figure S10. ^{1}H NMR spectrum of compound **3c** (DMSO-d_6, 500 MHz)

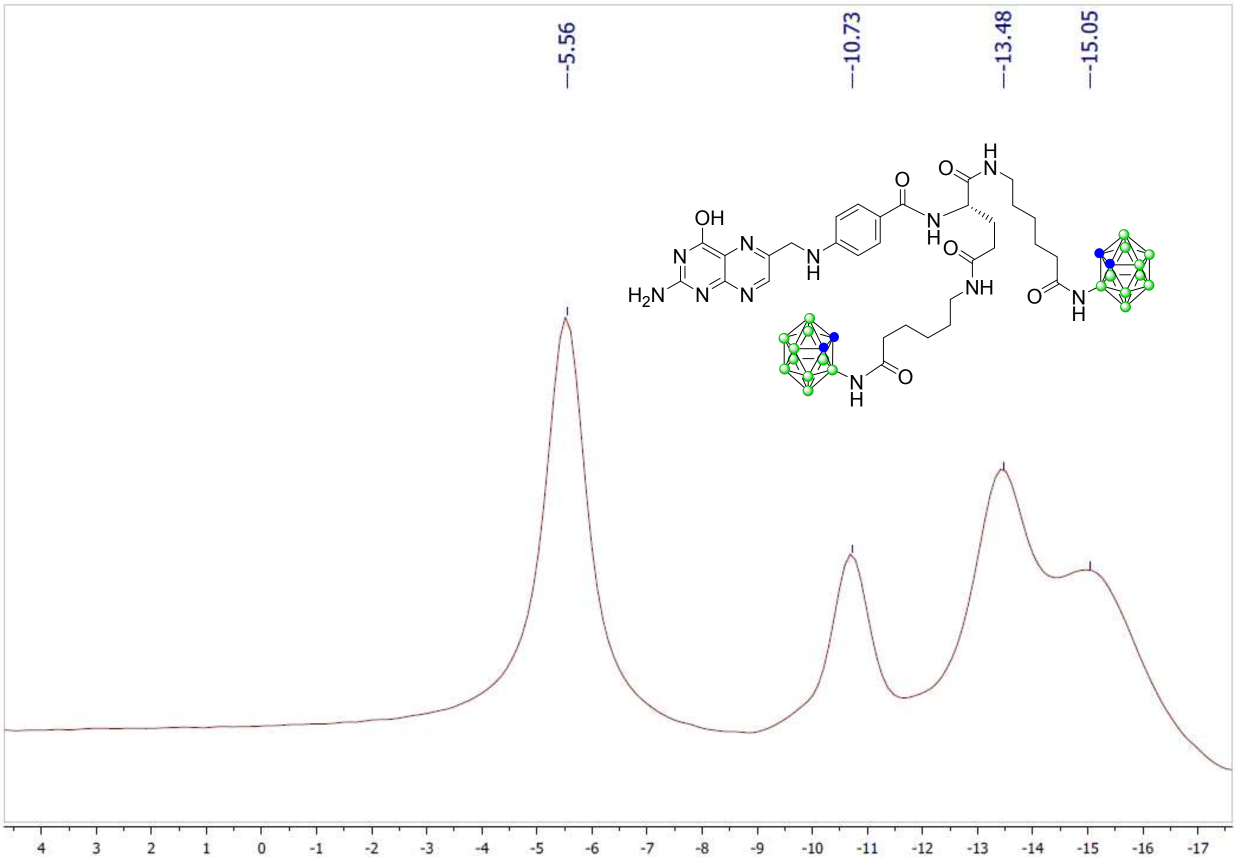

Figure S11. ^{11}B NMR spectrum of compound **3c** (DMSO-d_6, 160 MHz)

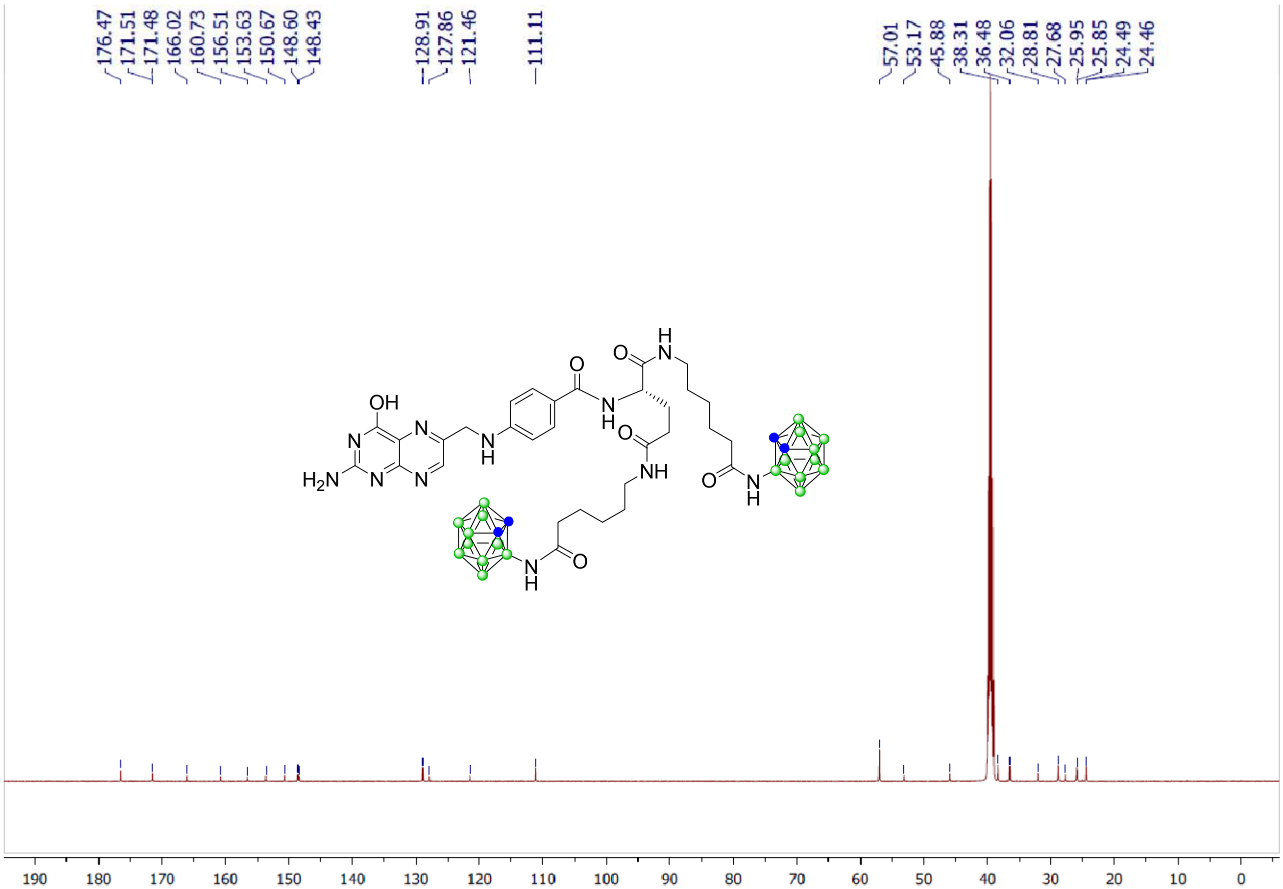

Figure S12. ^{13}C NMR spectrum of compound **3c** (DMSO-d_6, 126 MHz)

Table S1. Cell viability of various cell lines after 72-h co-incubation with compounds **3a** and **3b** and Cisplatin as a positive control[a]

Cell line	Compound	Concentration, mg/mL	Cell viability, %	SE, %[b]
BJ-5ta	**3a**	0.008	101.6	3.3
		0.016	102.8	2.5
		0.031	100.5	2.6
		0.063	104.0	0.9
		0.125	105.6	0.3
		0.250	99.2	2.6
		0.500	95.7	1.7
		1.000	76.2	2.1
	**3b**	0.008	110.6	0.8
		0.016	102.5	3.4
		0.031	99.2	5.5
		0.063	120.1	3.5
		0.125	136.9	4.1
		0.250	120.6	1.6
		0.500	87.9	3.1
		1.000	70.6	2.8
	**Cisplatin**	0.0008	87.5	1.6
		0.0016	86.9	4.5
		0.0031	82.3	2.0
		0.0063	79.7	1.9
		0.0125	71.3	3.2
		0.0250	44.9	3.7
		0.0500	28.5	2.6
		0.1000	18.5	0.2
DU 145	**3a**	0.008	96.3	4.6
		0.016	92.3	2.6
		0.031	89.6	2.9
		0.063	85.9	1.2
		0.125	80.7	2.8
		0.250	60.8	1.7
		0.500	40.0	1.9
		1.000	17.9	0.7
	**3b**	0.008	102.3	3.0
		0.016	101.3	5.9
		0.031	103.5	3.3
		0.063	110.5	8.3
		0.125	91.8	3.6
		0.250	79.9	3.2
		0.500	67.9	3.3
		1.000	19.8	1.4
	**Cisplatin**	0.0008	53.7	6.9
		0.0016	41.4	2.0
		0.0031	30.5	1.0
		0.0063	22.3	1.5
		0.0125	17.1	0.6
		0.0250	16.8	1.7
		0.0500	8.3	0.8
		0.1000	7.5	0.4

MDA-MB-231	**3a**	0.008	90.2	4.1
		0.016	87.7	2.2
		0.031	88.4	3.0
		0.063	85.6	2.8
		0.125	81.3	1.6
		0.250	60.0	2.6
		0.500	38.7	4.5
		1.000	30.7	2.5
	**3b**	0.008	95.0	2.5
		0.016	86.2	2.6
		0.031	86.8	1.3
		0.063	81.7	1.0
		0.125	76.0	1.8
		0.250	65.0	4.9
		0.500	43.3	2.3
		1.000	28.1	1.9
	**Cisplatin**	0.0008	85.0	4.1
		0.0016	77.3	2.4
		0.0031	71.0	2.9
		0.0063	55.9	5.8
		0.0125	33.4	1.6
		0.0250	20.5	1.1
		0.0500	13.9	0.4
		0.1000	12.8	0.5
SK-Mel-28	**3a**	0.008	104.7	1.6
		0.016	104.4	2.3
		0.031	104.4	2.2
		0.063	101.5	3.7
		0.125	101.5	3.5
		0.250	96.9	4.9
		0.500	91.1	2.6
		1.000	57.9	3.8
	**3b**	0.008	108.0	5.8
		0.016	109.8	5.0
		0.031	118.8	4.0
		0.063	133.1	3.6
		0.125	152.6	4.8
		0.250	121.4	5.4
		0.500	93.3	4.4
		1.000	59.1	3.6
	**Cisplatin**	0.0008	93.0	1.0
		0.0016	93.7	2.3
		0.0031	76.9	2.1
		0.0063	58.2	1.8
		0.0125	35.9	0.8
		0.0250	25.0	0.5
		0.0500	15.6	1.3
		0.1000	14.3	1.1

T98G	**3a**	0.008	103.5	7.1
		0.016	99.6	5.0
		0.031	92.4	2.2
		0.063	90.1	2.5
		0.125	81.3	2.6
		0.250	62.8	3.9
		0.500	32.8	1.4
		1.000	27.6	1.4
	**3b**	0.008	98.9	2.3
		0.016	99.4	2.6
		0.031	96.4	2.5
		0.063	104.2	2.8
		0.125	113.0	2.6
		0.250	103.1	1.4
		0.500	82.3	2.0
		1.000	48.0	1.5
	**Cisplatin**	0.0008	86.4	3.6
		0.0016	81.9	1.5
		0.0031	66.8	2.9
		0.0063	39.1	2.2
		0.0125	21.4	1.4
		0.0250	12.3	0.2
		0.0500	9.7	0.3
		0.1000	9.3	0.5
U87 MG	**3a**	0.008	66.8	2.1
		0.016	60.4	0.9
		0.031	50.8	3.4
		0.063	42.6	3.1
		0.125	46.5	2.2
		0.250	39.4	2.5
		0.500	31.3	1.2
		1.000	19.9	2.4
	**3b**	0.008	80.0	1.9
		0.016	74.1	5.8
		0.031	63.0	2.0
		0.063	53.5	2.3
		0.125	62.1	4.0
		0.250	60.6	2.2
		0.500	40.9	1.3
		1.000	19.1	1.9
	**Cisplatin**	0.0008	68.2	3.4
		0.0016	59.0	2.5
		0.0031	42.8	3.0
		0.0063	21.7	2.2
		0.0125	18.0	1.1
		0.0250	15.0	1.0
		0.0500	10.6	1.9
		0.1000	6.1	0.5

[a] Results of three independent experiments are presented

[b] SE is the standard error calculated according to equation: $SE = {}^{s}/_{\sqrt{3}}$, where *s* is the standard deviation

Boron Accumulation Assay

Boron accumulation in U87 MG, MDA-MB-231, SK-Mel-28, DU 145, T98G, and BJ-5ta cells was assessed after incubation in the presence of compounds **3a** and **3b** (0.25 mg/mL in the case of U87 MG cells; 0.5 mg/mL in other cases) for 10 min, 30 min, 1 h, 3 h, 6 h and 8 h. For analysis, 1 mL of the medium was taken, the number of cells was determined on a Countess automatic cell counter (Invitrogen, Waltham, MA, USA) and the boron content was analyzed using ICP-OES as described in [Tsygankova, A.R.; Kanygin, V.V.; Kasatova, A.I.; et al. Determination of boron by inductively coupled plasma atomic emission spectroscopy. Biodistribution of ^{10}B in tumour-bearing mice. *Russ. Chem. Bull.* **2020**, *69*, 601–607. https://doi.org/10.1007/s11172-020-2805-8]. The relative standard deviation (RSD) did not exceed 25% for the determination of boron by the ICP-AES method. Results of three independent experiments are presented.

Table S2. Boron accumulation by U87 MG human glioblastoma cells

Compound	Number of cells, ×10^6	Incubation time	Boron content in cells, μg B/10^6 cells
**3a**	3.0	10 min	0.01
			0.01
			0.01
	2.5	30 min	0.13
			0.15
			0.13
	2.0	1 h	0.12
			0.09
			0.09
	3.0	3 h	0.08
			0.07
			0.07
	3.0	6 h	0.10
			0.11
			0.10
	2.5	8 h	0.09
			0.07
			0.07
**3b**	1.0	10 min	0.63
			0.59
			0.58
	1.0	30 min	3.80
			3.60
			3.78
	1.0	1 h	3.22
			3.20
			3.90
	1.0	3 h	3.40
			4.17
			3.21
	1.0	6 h	5.50
			5.81
			5.80
	1.0	8 h	7.70
			6.62
			6.70

Table S3. Boron accumulation by MDA-MB-231 human breast carcinoma cells

Compound	Number of cells, $\times 10^6$	Incubation time	Boron content in cells, μg B/10^6 cells
**3a**	1.0	10 min	0.50
			0.53
			0.44
	0.7	30 min	0.74
			0.76
			0.80
	1.0	1 h	0.50
			0.46
			0.54
	1.0	3 h	0.42
			0.42
			0.37
	1.0	6 h	0.69
			0.73
			0.72
	1.4	8 h	0.46
			0.46
			0.51
**3b**	0.7	10 min	2.00
			2.14
			1.86
	0.9	30 min	1.67
			1.56
			2.11
	1.0	1 h	2.60
			2.50
			2.70
	1.0	3 h	1.40
			1.60
			1.40
	1.0	6 h	1.30
			1.40
			1.80
	1.0	8 h	2.00
			2.60
			3.00

Table S4. Boron accumulation by SK-Mel28 human melanoma cells

Compound	Number of cells, $\times10^6$	Incubation time	Boron content in cells, μg B/10^6 cells
**3a**	2.0	10 min	0.02
			0.02
			0.02
	0.9	30 min	0.04
			0.03
			0.04
	1.2	1 h	0.06
			0.06
			0.09
	0.8	3 h	0.08
			0.06
			0.07
	0.8	6 h	0.09
			0.02
			0.07
	1.3	8 h	0.05
			0.05
			0.04
**3b**	0.9	10 min	0.05
			0.07
			0.06
	1.2	30 min	0.13
			0.16
			0.13
	1.0	1 h	0.11
			0.11
			0.10
	0.9	3 h	0.28
			0.29
			0.26
	0.9	6 h	0.87
			1.07
			0.51
	2.0	8 h	0.15
			0.15
			0.14

Table S5. Boron accumulation by DU145 human prostate carcinoma cells

Compound	Number of cells, ×10^6	Incubation time	Boron content in cells, μg B/10^6 cells
**3a**	3.2	10 min	0.06
			0.05
			0.04
	2.8	30 min	0.10
			0.09
			0.08
	3.0	1 h	0.09
			0.10
			0.14
	2.4	3 h	0.27
			0.28
			0.28
	1.6	6 h	0.69
			0.65
			0.72
	3.4	8 h	0.22
			0.20
			0.20
**3b**	2.2	10 min	0.25
			0.24
			0.25
	2.8	30 min	0.20
			0.18
			0.18
	3.6	1 h	0.12
			0.12
			0.12
	2.4	3 h	0.31
			0.35
			0.34
	2.8	6 h	0.27
			0.28
			0.23
	3.0	8 h	0.42
			0.43
			0.42

Table S6. Boron accumulation by T98G human glioblastoma cells

Compound	Number of cells, ×10^6	Incubation time	Boron content in cells, μg B/10^6 cells
**3a**	2.8	10 min	0.08
			0.05
			0.05
	2.8	30 min	0.06
			0.06
			0.05
	1.3	1 h	0.22
			0.23
			0.22
	1.0	3 h	0.29
			0.36
			0.34
	1.1	6 h	0.34
			0.37
			0.35
	1.3	8 h	0.35
			0.39
			0.39
**3b**	2.8	10 min	0.14
			0.14
			0.10
	1.9	30 min	0.27
			0.31
			0.30
	1.0	1 h	0.89
			1.00
			0.89
	0.9	3 h	0.54
			0.54
			0.52
	1.4	6 h	0.61
			0.49
			0.56
	1.2	8 h	0.79
			0.81
			0.83

Table S7. Boron accumulation by BJ-5ta human foreskin fibroblasts

Compound	Number of cells, $\times 10^6$	Incubation time	Boron content in cells, μg B/10^6 cells
**3a**	1.0	10 min	0.03
			0.04
			0.08
	0.6	30 min	0.30
			0.17
			0.20
	1.5	1 h	0.05
			0.06
			0.04
	1.3	3 h	0.24
			0.24
			0.24
	1.8	6 h	0.10
			0.09
			0.09
	0.8	8 h	0.51
			0.50
			0.52
**3b**	0.7	10 min	0.76
			0.82
			0.86
	1.5	30 min	0.67
			0.74
			0.74
	0.9	1 h	0.82
			0.98
			0.98
	1.0	3 h	0.66
			0.75
			0.68
	1.8	6 h	0.28
			0.31
			0.37
	1.8	8 h	0.32
			0.33
			0.35
